# Supplementary material for: Drug repurposing for aging research using model organisms
Source: Aging Cell. 2017 Jun 16;16(5):1006–15. doi: 10.1111/acel.12626 (PMC5595691; doi:10.1111/acel.12626)
Supplement: Supplementary file 7 — Data S1 Zip‐Archive of all report cards. [file ACEL-16-1006-s007.zip › RC_9RA.pdf]

9RA

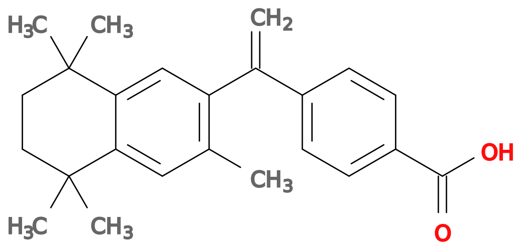

Database identifiers

ChEMBLCompound	CHEMBL1023
DrugBank	DB00307
CHEBI	50859
eMolecules	2794932

Ranking

	Rank	Score
Drosophila	344/697	0.436
C. elegans	NA	NA

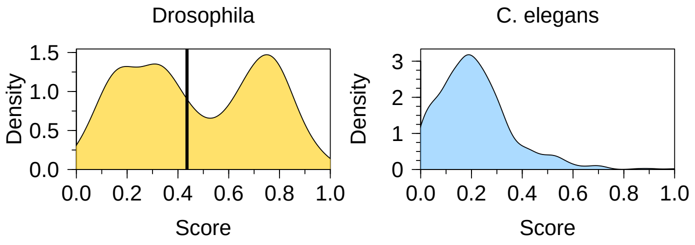

	Ageing implication	Domain conservation	Binding site conservation	Binding affinity	Bioavailability	Lipinski	Promiscuity	Purchasability	Drug approval	**Total**
Drosophila	0.624	0.649	0.841	0.933	(0.9)	-0.05	-0.0	0.1	0.1	0.436
C. elegans	NA	NA	NA	NA	NA	NA	NA	NA	NA	NA

Names

- **bexarotene**
- p-(1-(5,6,7,8-Tetrahydro-3,5,5,8,8-pentamethyl-2-naphthyl)vinyl)benzoic acid
- Targretin

Roles

antineoplastic agent, Retinoid X receptor agonist

Status

Approved drug (according to ChEMBL)	Yes
Administration Route	Oral, Topical
Number of Rule of 5 violations	1
Binding affinity to original target in log units (RF-Score prediction)	7.63
Burns *C. elegans* bioavailability prediction	-5.54

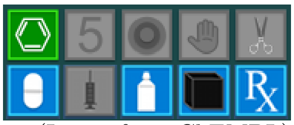

(Image from ChEMBL)

Medical Information

Indicatation: Used orally for the treatment of skin manifestations of cutaneous T-cell lymphoma (CTCL) in patients who are refractory to at least one prior systemic therapy. Also used topically for the treatment of skin lesions in early (stage IA and IB) CTCL in patients who experience refractory or persistent disease with the use of other therapies or are intolerant of other therapies.
Mechanism of action: Bexarotene selectively binds with and activates retinoid X receptor subtypes. There are three subtypes in total: RXR, RXR, RXR. The exact mechanism of action of bexarotene in the treatment of CTCL is unknown but the drug has activity in all clinical stages of CTCL.
Retail: Catalent Pharma Solutions, Contract Pharm, Eisai Inc.
Targretin 1% gel - 30.08 USD / g
Targretin 75 mg softgel - 36.54 USD / softget capsule
Targretin 75 mg capsule - 38.0 USD / capsule

(Information from DrugBank)

Compound Target Characteristics

Retinoic acid receptor RXR-alpha

Best gene implication in ageing for this target family came from gene Q0VJ96 via mapping the annotation from RGD 3610 annotated in RGD 2014-03-11. Annotation GO 7568 (aging) was Inferred from Expression Pattern

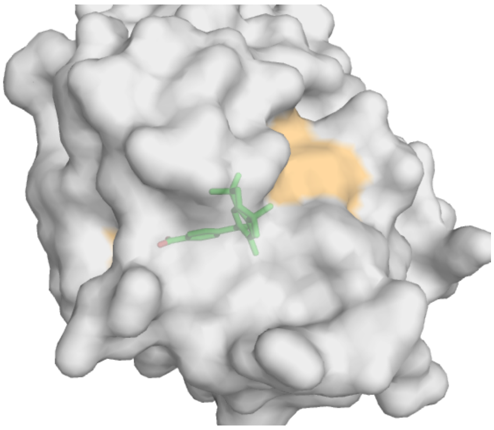

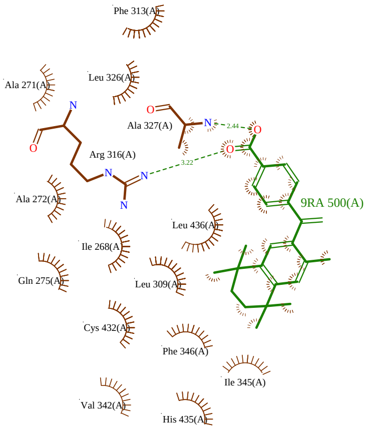

protein	amino acids contacts (binding site)
PDB:3h0a:chainA:P19793	I A A Q L F R L A V I F V C H L
PDB:4k6i:chainA:P19793	I A A Q L F R L A V I F V C H L
tr:B3KY83:B3KY83_HUMAN	I A A Q L F R L A V I F V C H L
sp:P19793:RXRA_HUMAN	I A A Q L F R L A V I F V C H L
tr:F1D8Q5:F1D8Q5_HUMAN	I A A Q L F R L A V I F V C H L
tr:Q6P3U7:Q6P3U7_HUMAN	I A A Q L F R L A V I F V C H L
tr:Q0VJ96:Q0VJ96_RAT	I A A Q L F R L A V I F V C H L
tr:Q6LC96:Q6LC96_MOUSE	I A A Q L F R L A V I F V C H L
sp:P28700:RXRA_MOUSE	I A A Q L F R L A V I F V C H L
tr:A2AJP1:A2AJP1_MOUSE	I A A Q L F R L A V I F V C H L
sp:P20153:USP_DROME	L V V Q L F R L N V I F I C H L

protein	whole protein ident	simil	domain-based ident	simil	contact-based ident	simil
PDB:3h0a:chainA:P19793	1.0	1.0	1.0	1.0	1.0	1.0
PDB:4k6i:chainA:P19793	1.0	1.0	1.0	1.0	1.0	1.0
tr:B3KY83:B3KY83_HUMAN	0.79	0.79	1.0	1.0	1.0	1.0
sp:P19793:RXRA_HUMAN	1.0	1.0	1.0	1.0	1.0	1.0
tr:F1D8Q5:F1D8Q5_HUMAN	1.0	1.0	1.0	1.0	1.0	1.0
tr:Q6P3U7:Q6P3U7_HUMAN	0.9	0.9	1.0	1.0	1.0	1.0
tr:Q0VJ96:Q0VJ96_RAT	0.97	0.99	1.0	1.0	1.0	1.0
tr:Q6LC96:Q6LC96_MOUSE	0.92	0.93	1.0	1.0	1.0	1.0
sp:P28700:RXRA_MOUSE	0.97	0.99	1.0	1.0	1.0	1.0
tr:A2AJP1:A2AJP1_MOUSE	0.97	0.99	1.0	1.0	1.0	1.0
sp:P20153:USP_DROME	0.37	0.61	0.4	0.66	0.69	0.84

usp (FBgn0003964) associated phenotypes

circadian rhythm defective, germline clone, heat sensitive, hypoactive, lethal - all die before end of pupal stage, lethal - all die during P-stage, lethal - all die during first instar larval stage, maternal effect, neuroanatomy defective, partially, rescuable maternal effect, somatic clone, some die during pupal stage, touch sensitivity defective

(Information from FlyBase)

usp (UniProt:P20153) annotation

Function: Receptor for ecdysone. May be an important modulator of insect metamorphosis. Plays an important part in embryonic and post-embryonic development. Binds to ecdysone response elements (ECRES) such as in the promoter region of s15 chorion gene.
Subunit: Heterodimer of USP and ECR. Only the heterodimer is capable of high-affinity binding to ecdysone. (PubMed:8247157).
Subcellular location: Nucleus.
Domain: Composed of three domains: a modulating N-terminal domain, a DNA-binding domain and a C-terminal ligand-binding domain.

(Information from UniProt)
